# Development and validation of a nomogram model for prediction of stroke-associated pneumonia associated with intracerebral hemorrhage

**DOI:** 10.1186/s12877-023-04310-5

**Published:** 2023-10-07

**Authors:** Ying Wang, Yuting Chen, Roumeng Chen, Yuchen Xu, Han Zheng, Jiajun Xu, Jinyang Xia, Yifan Cai, Huiqin Xu, Xinshi Wang

**Affiliations:** 1 Department of Neurology, The First Affiliated Hospital of Wenzhou Medical University, Wenzhou, Zhejiang China; 2 Graduate school, Wenzhou Medical University, Wenzhou, Zhejiang China; 3 First Clinical School of Medicine, Wenzhou Medical University, Wenzhou, Zhejiang China; 4 Key Laboratory of Alzheimer’s Disease of Zhejiang Province, Institute of Aging, Wenzhou Medical University, Wenzhou, Zhejiang China; 5 Department of Geriatrics, Geriatric Medical Center, The First Affiliated Hospital of Wenzhou Medical University, Wenzhou, Zhejiang China

**Keywords:** Intracranial hemorrhage, Stroke-associated pneumonia, Predictors, Nomogram, Validation

## Abstract

**Background:**

We aimed to establish risk factors for stroke-associated pneumonia (SAP) following intracerebral hemorrhage (ICH) and develop an efficient and convenient model to predict SAP in patients with ICH.

**Methods:**

Our study involved 1333 patients consecutively diagnosed with ICH and admitted to the Neurology Department of the First Affiliated Hospital of Wenzhou Medical University. The 1333 patients were randomly divided (3:1) into the derivation cohort (n = 1000) and validation Cohort (n = 333). Variables were screened from demographics, lifestyle-related factors, comorbidities, clinical symptoms, neuroimaging features, and laboratory tests. In the derivation cohort, we developed a prediction model with multivariable logistic regression analysis. In the validation cohort, we assessed the model performance and compared it to previously reported models. The area under the receiver operating characteristic curve (AUROC), GiViTI calibration belt, net reclassification index (NRI), integrated discrimination index (IDI) and decision curve analysis (DCA) were used to assess the prediction ability and the clinical decision-making ability.

**Results:**

The incidence of SAP was 19.9% and 19.8% in the derivation (n = 1000) and validation (n = 333) cohorts, respectively. We developed a nomogram prediction model including age (Odds Ratio [OR] 1.037, 95% confidence interval [CI] 1.020–1.054), male sex (OR 1.824, 95% CI 1.206–2.757), multilobar involvement (OR 1.851, 95% CI 1.160–2.954), extension into ventricles (OR 2.164, 95% CI 1.456–3.215), dysphagia (OR 3.626, 95% CI 2.297–5.725), disturbance of consciousness (OR 2.113, 95% CI 1.327–3.362) and total muscle strength of the worse side (OR 0.93, 95% CI 0.876–0.987). Compared with previous models, our model was well calibrated and showed significantly higher AUROC, better reclassification ability (improved NRI and IDI) and a positive net benefit for predicted probability thresholds between 10% and 73% in DCA.

**Conclusions:**

We developed a simple, valid, and clinically useful model to predict SAP following ICH, with better predictive performance than previous models. It might be a promising tool to assess the individual risk of developing SAP for patients with ICH and optimize decision-making.

**Supplementary Information:**

The online version contains supplementary material available at 10.1186/s12877-023-04310-5.

## Background

Stroke-associated pneumonia (SAP) was defined as pneumonia complicating the first 7 days after stroke onset in nonventilated patients [1]. As a frequently encountered complication after stroke, SAP developed in 6.5-33% patients with stroke [2–7]. SAP increases the economic burden on stroke patients [8], lengthens the hospitalization duration, and is associated with a poor prognosis by increasing the risk of a negative and fatal outcome [9]. Therefore, prompt identification of risk factors associated with SAP and making preventive interventions may help reduce the risk of developing SAP and thus benefiting the patients.

Several risk factors related to the development of SAP have been identified in previous studies. These include older age, male, hypertension, diabetes, atrial fibrillation [10], previous history of chronic obstructive pulmonary disease (COPD) [11], dysphagia [12], pre-stroke dependence [11], intracerebral hemorrhage (ICH) [13], higher admission National Institutes of Health Stroke Scale (NIHSS) score [6], lower Glasgow Coma Scale score (GCS) [14], infratentorial location [11], extension of hemorrhage into ventricles [11], hematoma volume [11], stroke-induced immunodepression syndrome [12], and increased C-reactive protein [15]. Based on these risk factors, some prediction models or scoring systems were constructed to help identify patients at elevated risk of developing SAP [6, 7, 10, 11, 14].

Studies have shown that patients with ICH have a higher risk of developing SAP (8.5-16.9%) [7, 11]compared to patients with ischemic stroke (6.5-12.2%) [6, 7, 10, 13, 14]. However, most of these studies only included patients with ischemic stroke. There are few studies on SAP following ICH. One is ISAN score, which was developed from a multi-center registry including both patients with ischemic stroke and ICH and incorporated parameters including pre-stroke dependence, sex, age, and NIHSS score [7]. Because this model does not include ICH-specific variables, it did not perform well in ICH patients, with a AUROC of 0.71 (0.66 to 0.77) compared to 0.78 (0.76 to 0.80) in the patients with ischemic stroke [7]. Another one was ICH-associated pneumonia score (ICH-APS). It developed two prediction models with or without hematoma volume as an included variable (ICH-APS-B and ICH-APS-A, respectively). Other variables in the prediction models included age, current smoking, excess alcohol consumption, COPD, pre-stroke dependence, admission GCS score, admission NIHSS score, dysphagia, location of ICH, and intraventricular extension [11]. This scoring model is relatively complex, in which the GCS and NIHSS scores overlap to some extent and may have collinearity. Different parts of the NIHSS score have different impacts on SAP development. For example, paralysis and dysphasia probably contributed more to SAP development than ataxia and loss of visual field. Direct use of NIHSS score without considering the specific factors may weaken some critical factors’ influence on SAP.

We aimed to establish risk factors for SAP following ICH from more specific and simplified clinical variables and to build a more efficient and convenient model to predict SAP in patients with ICH.

## Methods

### Study design and source of data

This retrospective cohort study involved 1333 patients consecutively diagnosed with ICH and admitted to the Neurology Department of the First Affiliated Hospital of Wenzhou Medical University from January 2010 to December 2019. Primary inclusion criteria for the study were adult patients (aged ≥ 18 years) diagnosed with ICH confirmed by head CT imaging [16]. Exclusion criteria included (1) crucial clinical data incomplete or missing (e.g., patients without an initial CT performed within 72 h post-ICH); (2) lung infection developed before onset of ICH; (3) severe mental or cognitive impairment before ICH and unable to cooperate with the examination.

We sequentially numbered the 1333 patients included according to the admission date and designated it as the overall cohort dataset. Using the “train_test_split” method in Python, we randomly divided the overall cohort dataset into derivation and validation cohorts at a ratio of 3:1, with 1000 patients in the derivation cohort and 333 patients in the validation cohort. This study obtained approval by the Ethics Committee of the First Affiliated Hospital of Wenzhou Medical University (No.2020 − 185). Informed consent was obtained verbally through telephone interviews with the participants or their legal representatives and how their data will be collected, used, and protected were explained to them. To protect privacy, patients’ identifying information (e.g., names, addresses) were removed from the dataset throughout the research to ensure participants’ anonymity and the data is stored securely using encryption and access controls.

### Variables

We collected data from the electronic medical records system of the First Affiliated Hospital of Wenzhou Medical University at the time of initial admission, including demographics, life style-associated factors, comorbidities, clinical symptoms, neuroimaging characteristics, and laboratory examinations. Demographic variables included sex aand age at the time of ICH. Lifestyle factors included current smoking and alcohol intake status. The subject’s medical history was notable for comorbidities that comprised hypertension, diabetes, hyperlipidemia, ischemic heart disease, hyperuricemia, and COPD. Variables associated with the clinical symptoms included pre-stroke dependence (modified Rankin Scale [mRS] ≥ 2), presence of dysphagia (assessed with a swallow test by a physical therapist), the status of consciousness (classified as sopor, somnolence or coma), total muscle strength of the worse side (ranging from 0 to10), vomiting after ICH, admission GCS and NIHSS. As for the status of consciousness, somnolence is characterized as a state of strong desire for sleep, or sleeping for unusually long periods but can be arousable by minor stimulation to obey, answer, or respond. Sopor is defined as a condition of abnormally deep sleep that the patients can only be arousable by repeated strong or painful stimulation to respond. Coma is defined as a deep state of prolonged unconsciousness in which a person cannot be awakened, fails to respond normally to stimuli or exhibits reflex responses.

In addition, neuroimaging characteristics of ICH were recorded, including multilobar involvement, deep region involvement, extension of hemorrhage into ventricles, and lesion volume. Volumetric assessment of the lesion location was determined using the ABC/2 method [17]. Based on previous research indicating that hematoma volume greater than 20ml is an important factor associated with poor prognosis [18, 19], we categorized hematoma volume into two groups using 20ml as the boundary. Laboratory examinations at initial admission included red blood cells, platelet, albumin, blood glucose, and creatinine.

The predictive outcome of our study was whether patients developed SAP following ICH. SAP was defined as a range of pulmonary infections affecting the lower respiratory tract that developed within the first 7 days after stroke onset according to the recommendations of the Pneumonia in Stroke Consensus Group. It was diagnosed on a basis of clinical and laboratory indices of respiratory tract infection (e.g., fever, new purulent sputum, cough, bronchial breath sounds or worsening gas exchange), and supported by typical chest radiographs findings [1].

### Model development and validation

We randomly divided the study cohort into a derivation and validation cohort at a 3:1 ratio. The candidate variables in the derivation cohort were screened for collinearity using the linear regression test. Variance inflation factor (VIF) > 5 was considered the existence of collinearity. We performed a univariable analysis of the candidate variables in the derivation cohort with simple logistic regression and chose variables with P-value < 0.2 for multivariable analysis. A total of 16 predictive variables were entered into the multivariable logistic regression for model development using the forward stepwise method based on likelihood ratio test. A nomogram model for prediction was constructed, based on the findings of the logistic regression, with the model’s goodness of fit assessed using the Hosmer-Lemeshow test.

In the validation cohort, we assessed the model performance and compared it to previously reported prediction models (details of these baseline models were displayed in Supplementary Tables 2,3) with the area under the receiver operating characteristic curve (AUROC) and accuracy (GiViTI calibration belt), decision curve analysis (DCA)、net reclassification index (NRI) and integrated discrimination index (IDI). The GiViTI calibration belt reveals the relationship between predicted and observed probabilities, including 80% and 95% confidence intervals [20, 21]. DCA was employed to assess the clinical utility, especially the capacity to enhance decision-making, of the prediction models by quantifying the net benefits at various threshold probabilities [22]. NRI and IDI reflect the ability of a new model to appropriately reassign people into different risk strata [23].

### Statistical analysis

Continuous variables, such as age and total muscle strength on the worse side, were evaluated in terms of mean ± standard deviation (SD) or median and interquartile range (IQR) as appropriate, while categorical variables were expressed as counts and percentages. Univariable analysis of candidate variables was performed using univariate logistic regression. Variables with a two-sided P-value < 0.2 were entered into the multivariate logistic regression to build the prediction model using the forward stepwise method. The multivariable analysis results were presented using a Forest plot with the ‘forest’ package. P-value < 0.05 was considered statistically significant in multivariate logistic regression and the GiViTI calibration test. Univariable analysis and multivariable logistic regression were performed using IBM SPSS Statistics 25.0 software (IBM Corporation, NY, USA). The AUROC, GiViTI calibration belt, DCA analyses, NRI and IDI were performed using R version 4.2.1 (R Project for Statistical Computing) with the pROC, givitiR, rmda, and PredictABEL libraries (http://lib.stat.cmu.edu/R/CRAN/).

During statistical analysis, missing data of hematoma volume (22/1333,1.7%) and blood glucose (3/1333,0.23%) were considered missing completely at random (MCAR) [9]. As the missing data was less than 5%, we employed pairwise deletion, an indirect (passive) method, to maximize data utilization.

## Results

A total of 1350 patients diagnosed with ICH met the primary inclusion criteria. After reviewing the medical records, we excluded 12 patients without an initial CT performed within 72 h post-ICH, one patient who had severe mental or cognitive disorders before ICH and could not cooperate with the examination, and 4 patients whose lung infection developed before the cerebral hemorrhage. Ultimately, 1333 eligible participants were included in the study for analysis, including 1000 patients in the derivation cohort and 333 patients in the validation cohort (Table 1; Fig. 1).

**Table 1:** Baseline characteristics

Variables		Overall Cohort N = 1333	Derivation Cohort n = 1000	Validation Cohort n = 333	p-value	
**Demographics**						
Age (years), median (IQR)		60 (52–68)	60 (52–68)	61 (52–68)	0.693	
Sex (male), No./total No. (%)		926/1333 (69.5)	706/1000 (70.6)	220/333 (66.1)	0.120	
**Life style-associated factors**						
Smoking, No./total No. (%)		534/1333 (40.1)	405/1000 (40.5)	129/333 (38.7)	0.570	
Alcohol drinking, No./total No. (%)		516/1333 (38.7)	381/1000 (38.1)	135/333 (40.5)	0.428	
**Comorbidities**						
Hypertension, No./total No. (%)		1219/1333 (91.4)	916/1000 (91.6)	303/333 (91.0)	0.731	
Diabetes, No./total No. (%)		262/1333 (19.7)	192/1000 (19.2)	70/333 (21.0)	0.469	
Hyperlipidemia, No./total No. (%)		275/1333 (20.6)	196/1000 (19.6)	79/333 (23.7)	0.107	
Ischemic heart disease, No./total No. (%)		42/1333 (3.2)	27/1000 (2.7)	15/333 (4.5)	0.103	
Hyperuricemia, No./total No. (%)		81/1333 (6.1)	53/1000 (5.3)	28/333 (8.4)	0.040	
COPD, No./total No. (%)		33/1333 (2.5)	26/1000 (2.6)	7/333 (2.1)	0.613	
**Clinical symptoms**						
Prestroke dependence (mRS ≥ 2), No./total No. (%)		43/1333 (3.2)	35/1000 (3.5)	8/333 (2.4)	0.326	
Dysphagia, No./total No. (%)		257/1333 (19.3)	195/1000 (19.5)	62/333 (18.6)	0.724	
Disturbance of consciousness, No./total No. (%)		317/1333 (23.8)	230/1000 (23.0)	87/333 (26.1)	0.246	
alert		1016/1333(76.2)	770/1000 (77.0)	246/333 (73.8)		
somnolence		209/1333(15.7)	143/1000 (14.3)	66/333 (19.8)		
sopor		88/1333(6.6)	71/1000 (7.1)	17/333(5.1)		
coma		20/1333(1.5)	16/1000 (1.6)	4/333(1.2)		
Total muscle strength of worse side, median (IQR)		8 (3–8)	8 (4–8)	7 (3–8)	0.150	
Vomiting after ICH, No./total No. (%)		279/1333 (20.9)	201/1000 (20.1)	78/333 (23.4)	0.197	
NIHSS#		6 (2–11)	6 (2–11)	7 (2–11)	0.060	
GCS		15 (14–15)	15 (14–15)	15 (14–15)	0.617	
**Neuroimaging characteristics**						
Multilobar involvement, No./total No. (%)		201/1333 (15.1)	138/1000 (13.8)	63/333 (18.9)	0.024	
Deep region involvement, No./total No. (%)		1023/1333 (76.7)	759/1000 (75.9)	264/333 (79.3)	0.206	
Extension into ventricles, No./total No. (%)		291/1333 (21.8)	222/1000 (22.2)	69/333 (20.7)	0.571	
Lesion volume (ml), median (IQR)		7.26 (3.01–14.82)	7.32 (3.02–14.63)	7.00 (3.01–15.12)	0.709	
**Laboratory Examinations**						
Red blood cell (10^12^/L), median (IQR)		4.55 (4.24–4.91)	4.56 (4.24–4.92)	4.55 (4.25–4.89)	0.536	
Platelet (10^9^/L), median (IQR)		204 (169–244)	204 (169–245)	204 (168–244)	0.996	
Albumin, median (g/L) (IQR)		40.15 (37.6–42.7)	40.25 (37.6–42.7)	40.1 (37.9–42.7)	0.762	
Blood glucose (mmol/L), median (IQR)		5.7 (4.9–6.9)	5.7 (4.9–6.9)	5.8 (4.9–7.2)	0.292	
Creatinine (μmol/L), median (IQR)		67 (56–80)	67 (56–80)	67 (56–81)	0.875	

Continuous variables that exhibited normal distribution were presented as mean (standard deviation [SD]); non-normal variables were presented as median (interquartile range [IQR]); quantitative variables were presented as number/total number (%).

SAP: stroke-associated pneumonia; COPD:chronic obstructive pulmonary disease; mRS : modified Rankin scale; ICH:intracerebral hemorrhage; NIHSS: National Institutes of Health Stroke Scale; GCS: Glasgow Coma Scale score;

#VIF = 6.899, exclude from further analysis

**Fig. 1:**
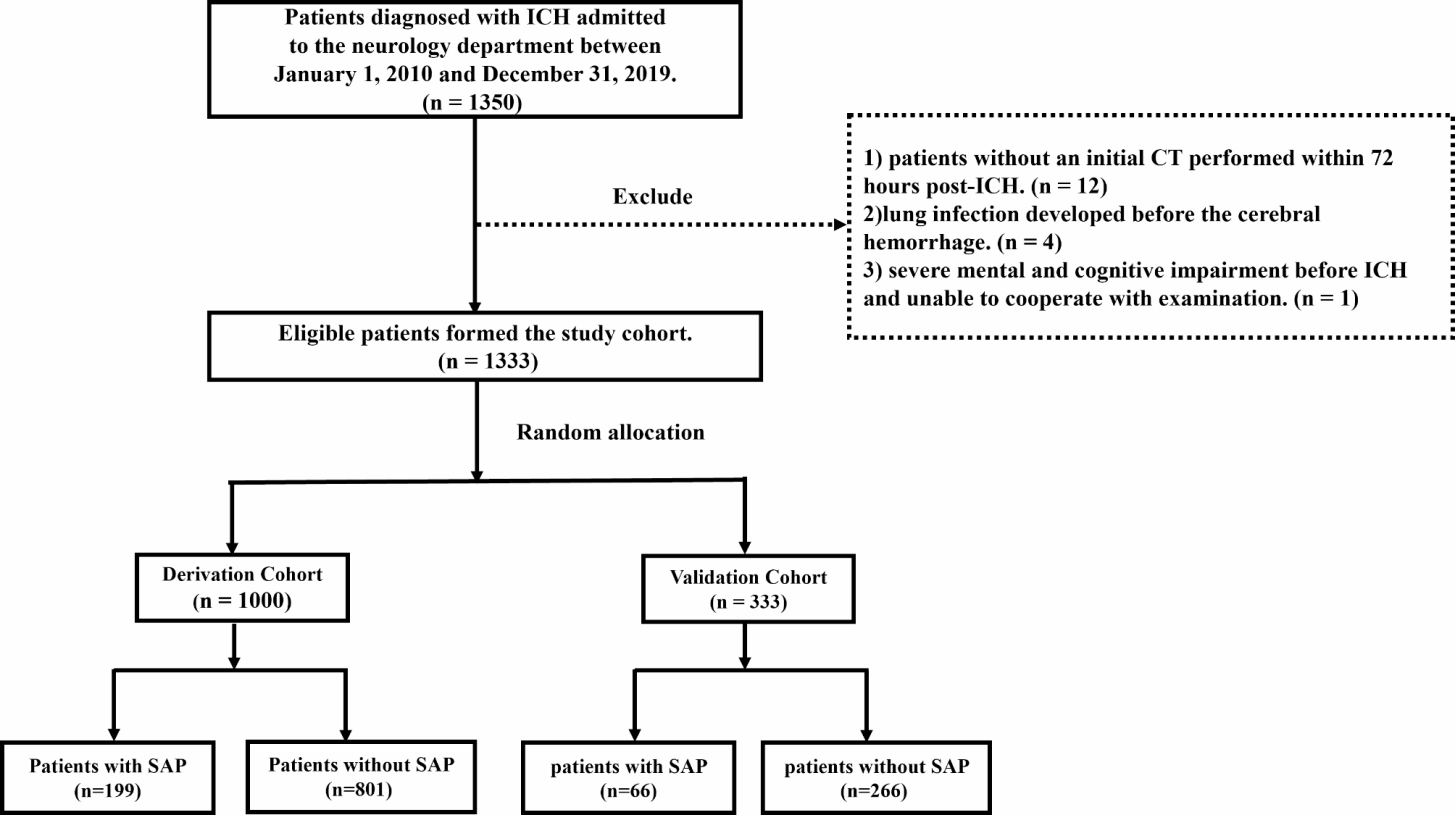
Flow chart of patient recruitment. Flow-chart concisely outlines sequential application of inclusion and exclusion criteria that ultimately lead to the definition of the final study cohort SAP: stroke-associated pneumonia, ICH: intracerebral hemorrhage

### Characteristics and univariable analysis of the derivation cohort

Table 2 displays the characteristics of the study cohort. The derivation cohort comprised 199 (19.9%, n = 199/1000) patients with SAP and 801 (80.1%, n = 801/1000) without SAP. The median age of patients with SAP is 62 (IQR 55–72), which was significantly older than those without SAP (median 59, IQR 52–68, p < 0.001, 95%CI 1.016–1.045). Patients with SAP exhibited a higher likelihood of experiencing impaired consciousness (50.8%, n = 101/199 vs. 16.1%, n = 129/801, p < 0.001, 95%CI 3.838–7.511) and dysphagia (49.7%, n = 99/199 vs. 12.0%, n = 96/801, p < 0.001, 95%CI 5.122–10.320) compared to those without SAP. Patients with SAP demonstrated a significantly lower median total muscle strength on the worse side compared to those without SAP (median 5, IQR 0–8 vs. median 8, IQR 5–9, p < 0.001, 95%CI 0.791–0.866). Multilobar involvement (23.6%, n = 47/199 vs. 11.4%, n = 91/801, p < 0.001, 95%CI 1.628–3.575), lesion volume ≥ 20mL (25.0%, n = 49/196 vs. 11.1%, n = 87/786, p < 0.001, 95%CI 1.808–3.966) and extension of hemorrhage into ventricles (36.2%, n = 72/199 vs. 18.7%, n = 150/801, p < 0.001, 95%CI 1.753–3.454) occurred more frequently in patients with SAP compared to those without SAP. Pre-stroke dependence was present in 7.0% (n = 14/199) of patients with SAP, while only 2.6% (n = 21/801) of those without SAP (p = 0.004, 95%CI 1.403–5.632). In the GCS score, patients with SAP had a greater probability of scoring ≤ 14 than those without SAP (55.3%, n = 110/199 vs. 21.2%, n = 170/801, p < 0.001, 95%CI 3.309–6.361). In addition, patients with SAP exhibited markedly elevated levels of blood glucose and creatinine compared to those without SAP.

**Table 2:** Univariable analysis

Variables	Derivation Cohort n = 1000			Validation Cohort n = 333		
	patients with SAP n = 199	patients without SAP n = 801	p-value	patients with SAP n = 66	patients without SAP n = 267	p-value
**Demographics**						
Age (years), median (IQR)	62 (55–72)	59 (52–68)	< 0.001*	64 (53–72)	60 (52–67)	0.035*
Sex (male), No./total No. (%)	149/199 (74.9)	557/801 (69.5)	0.140	43/66 (65.2)	177/267 (66.3)	0.861
**Life style-associated factors**						
Smoking, No./total No. (%)	90/199 (45.2)	315/801 (39.3)	0.130	27/66 (40.9)	102/267 (38.2)	0.686
Alcohol drinking, No./total No. (%)	81/199 (40.7)	300/801 (37.5)	0.398	26/66 (39.4)	109/267 (40.8)	0.832
**Comorbidities**						
Hypertension, No./total No. (%)	180/199 (90.5)	736/801 (91.9)	0.515	58/66 (87.9)	245/267 (91.8)	0.327
Diabetes, No./total No. (%)	38/199 (19.1)	154/801 (19.2)	0.967	11/66 (16.7)	59/267 (22.1)	0.334
Hyperlipidemia, No./total No. (%)	35/199 (17.6)	161/801 (20.1)	0.425	6/66 (9.1)	73/267 (27.3)	0.003*
Ischemic heart disease, No./total No. (%)	7/199 (3.5)	20/801 (2.5)	0.429	5/66 (7.6)	10/267 (3.7)	0.188
Hyperuricemia, No./total No. (%)	10/199 (5.0)	43/801 (5.4)	0.847	6/66 (9.1)	22/267 (8.2)	0.823
COPD, No./total No. (%)	8/199 (4.0)	18/801 (2.2)	0.165	3/66 (4.5)	4/267 (1.5)	0.142
**Clinical symptoms**						
Prestroke dependence (mRS ≥ 2), No./total No. (%)	14/199 (7.0)	21/801 (2.6)	0.004*	3/66 (4.5)	5/267 (1.9)	0.219
Dysphagia, No./total No. (%)	99/199 (49.7)	96/801 (12.0)	< 0.001*	36/66 (54.5)	26/267 (9.7)	< 0.001*
Disturbance of consciousness, No./total No. (%)	101/199 (50.8)	129/801 (16.1)	< 0.001*	34/66 (51.5)	53/267 (19.9)	< 0.001*
Total muscle strength of worse side, median (IQR)	5 (0–8)	8 (5–9)	< 0.001*	5 (0–8)	7 (3–8)	0.002*
Vomiting after ICH, No./total No. (%)	46/199 (23.1)	155/801 (19.4)	0.236	17/66 (25.8)	61/267 (22.8)	0.617
GCS(≤ 14 )	110/199 (55.3)	170/801 (21.2)	< 0.001*	35/66 (53.0)	57/267 (21.3)	< 0.001*
**Location of intracerebral hemorrhage**						
Multilobar involvement, No./total No. (%)	47/199 (23.6)	91/801 (11.4)	< 0.001*	21/66 (31.8)	42/267 (15.7)	0.003*
Deep region involvement, No./total No. (%)	160/199 (80.4)	599/801 (74.8)	0.098	51/66 (77.3)	213/267 (79.8)	0.654
Extension into ventricles, No./total No. (%)	72/199 (36.2)	150/801 (18.7)	< 0.001*	27/66 (40.9)	42/267 (15.7)	< 0.001*
Lesion volume(≥ 20mL), No./total No. (%)	49/196 (25.0)	87/786 (11.1)	< 0.001*	20/64 (31.3)	31/265 (11.7)	< 0.001*
**Laboratory Examinations**						
Red blood cell (< 3.8 10^12^/L ), No./total No. (%)	15/199 (7.5)	48/801 (6.0)	0.423	7/66 (10.6)	12/267 (4.5)	0.063
Platelet(< 125 10^9^/L ), No./total No. (%)	13/199 (6.5)	40/801 (5.0)	0.387	4/66 (6.1)	17/267 (6.4)	0.927
Albumin(< 40 g/L), No./total No. (%)	103/199 (51.8)	371/801 (46.3)	0.169	32/66 (48.5)	126/267 (47.4)	0.871
Blood glucose (> 6.1mmol/L), No./total No. (%)	96/199 (48.2)	273/798 (34.2)	< 0.001*	35/66 (53.0)	103/267 (38.6)	0.034*
Creatinine(> 97μmol/L), No./total No. (%)	26/199 (13.1)	62/801 (7.74)	0.019*	9/66 (13.6)	20/267 (7.5)	0.118

Continuous variables that exhibited normal distribution were presented as mean (standard deviation [SD]); non-normal variables were presented as median (interquartile range [IQR]); quantitative variables were presented as number/total number (%)

SAP: stroke-associated pneumonia; COPD:chronic obstructive pulmonary disease; mRS : modified Rankin scale; ICH:intracerebral hemorrhage; GCS: Glasgow Coma Scale score;

### Multivariable analysis and construction of prediction model for SAP

**Fig. 2:**
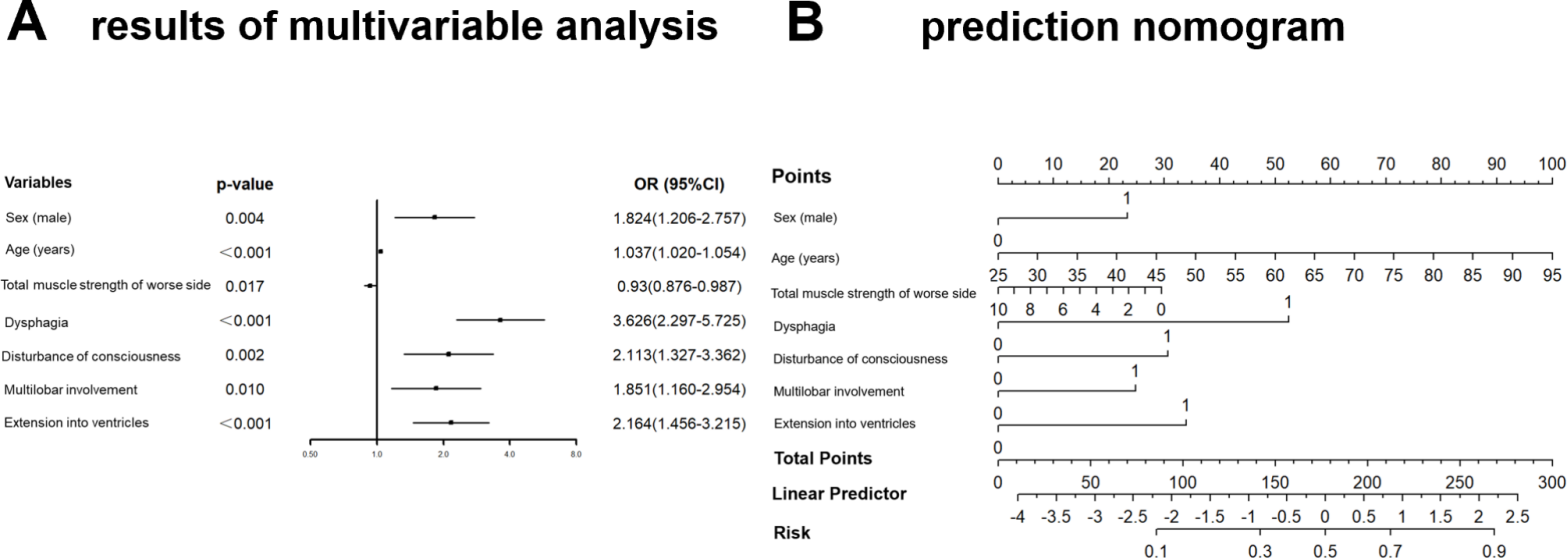
Prediction of SAP probability using results of multivariable analysis. **A**, Forest plot based on the results of multivariable analysis. **B**, Prediction nomogram based on the results of the multivariable analysis conducted on the entire cohort OR: Odds Ratio, 95% CI: 95% confidence interval

A total of 16 variables with p < 0.2 were entered into the multivariate logistic analysis, among which NHISS was excluded from further analysis due to the existence of collinearity (VIF = 6.899, Supplementary Table 1). These included age, sex (male), smoking, COPD, pre-stroke dependence (mRS ≥ 2), dysphagia, disturbance of consciousness, total muscle strength of the worse side, GCS (≤ 14), multilobar involvement, deep region involvement, extension into ventricles, lesion volume(≥ 20mL), albumin(< 40 g/L), blood glucose (> 6.1mmol/L) and creatinine(> 97μmol/L). The outcomes of the multivariable logistic analysis are shown in Fig. 2A. Variables retained in our model included age (Odds Ratio [OR] 1.037, 95% confidence interval [CI] 1.020–1.054, P < 0.001), sex (OR 1.824, 95% CI 1.206–2.757, P = 0.004), multilobar involvement (OR 1.851, 95% CI 1.160–2.954, P = 0.01 ), extension into ventricles (OR 2.164, 95% CI 1.456–3.215, P < 0.001), dysphagia (OR 3.626, 95% CI 2.297–5.725, P < 0.001), disturbance of consciousness (OR 2.113, 95% CI 1.327–3.362, P = 0.002) and total muscle strength of the worse side (OR 0.93, 95% CI 0.876–0.987, P = 0.017). Upon analyzing the data using logistic regression, we have developed a nomogram that predicts the individualized risk of developing SAP during hospitalization (Fig. 2B). The AUROC of the model was 0.793. The Hosmer–Lemeshow test was not significant(P = 0.203).

The application of the nomogram was as follows: based on the nomogram, we first calculate the score for each prediction indicator and then sum them to obtain the total score. The resulting total score can be used to estimate the individualized risk of developing SAP during hospitalization. For example, a male patient (24 points) was 70-year-old (63 points), had a disorder of consciousness (30 points), and had the symptom of dysphagia (52 points). Total muscle strength of the worse side was 0 (30 points), and only one lobe was involved (0 points). The hematoma didn’t extend into the ventricle (0 points). The cumulative score of the prediction indicators was 24 + 63 + 30 + 52 + 30 + 0 + 0 = 199 points, and the corresponding predicted risk for him to develop SAP was about 60% (Fig. 2B).

### Evaluation and validation of model performance

**Fig. 3:**
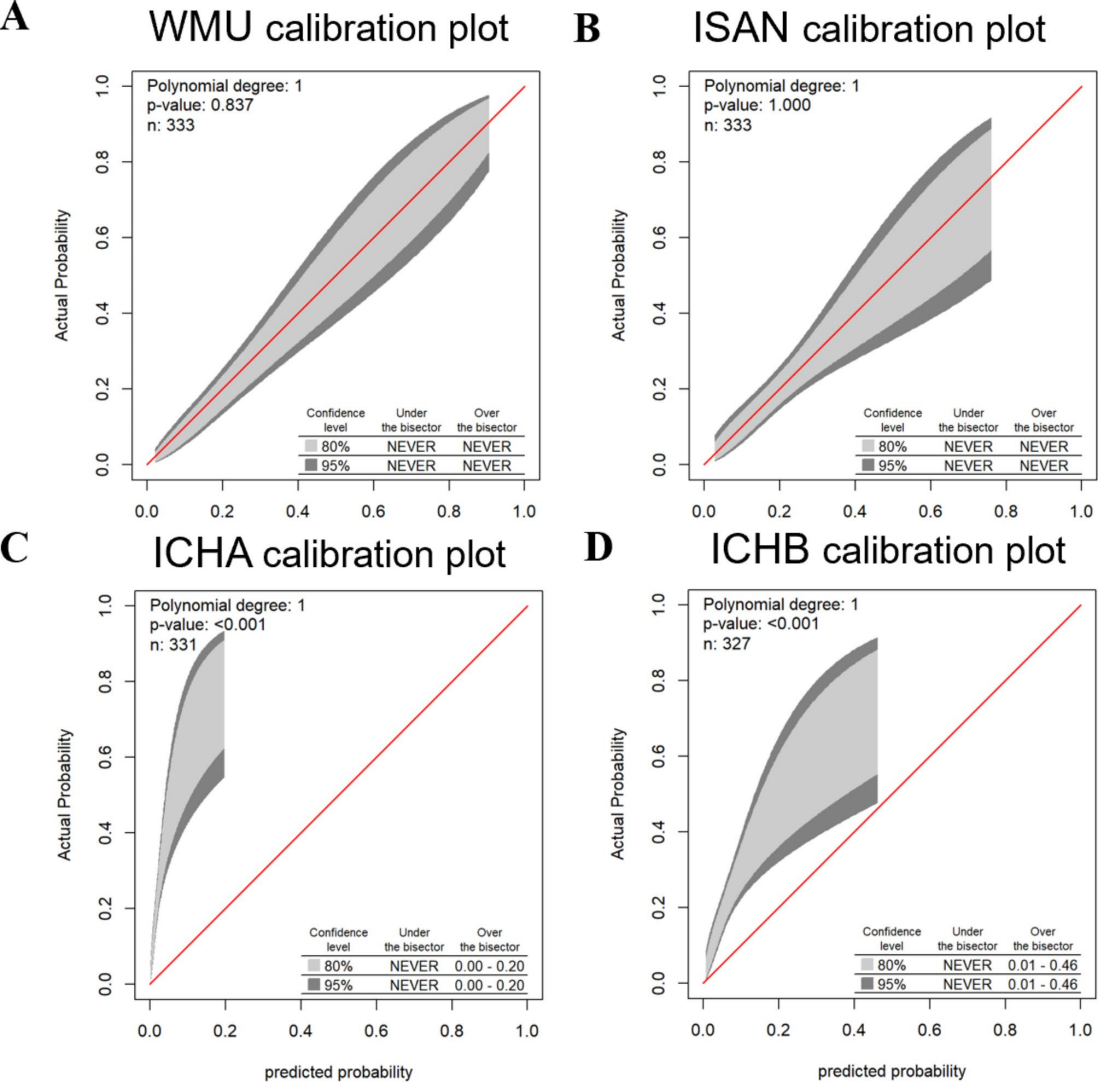
Calibration Belts in the Validation cohort. (**A**) our prediction model, (**B**) the ISAN model, (**C**) the ICH-A model, (**D**) the ICH-B model. The calibration bands with 80% and 95% confidence levels are shown in light and dark gray, respectively. The bottom-right table shows the predicted probability ranges. P > 0.05 were deemed that the model fit was good

As shown in Fig. 3, the GiviTI calibration belt demonstrated that our and ISAN models were well calibrated with no significant deviation between the predicted and actual probabilities (P = 0.837 and P = 1.000, respectively). However, the ICH-A and ICH-B models showed a significant dissimilarity between the predicted and actual probabilities (P < 0.001 and P < 0.001, respectively), which means these models were not well calibrated.

**Fig. 4:**
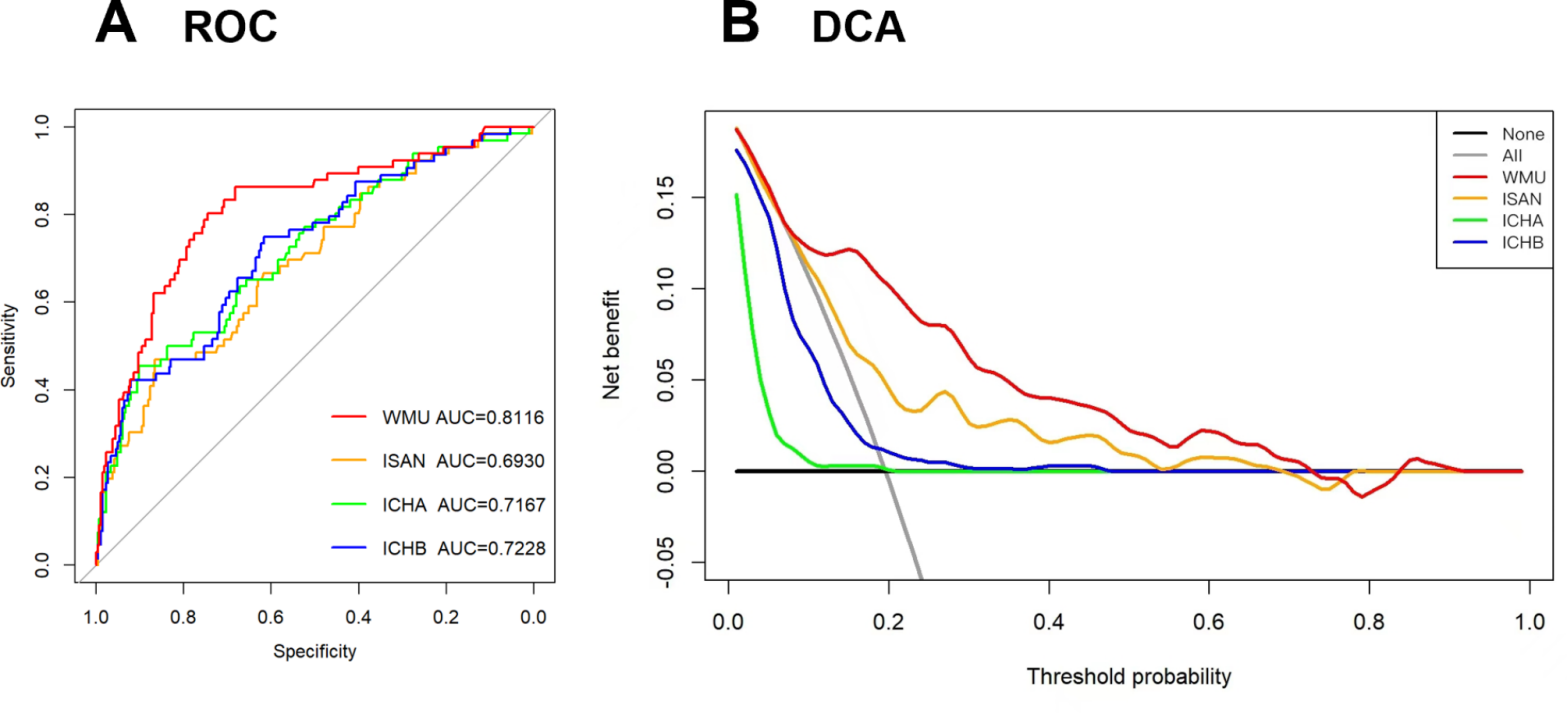
ROC and decision curves in the Validation cohort. **A**, ROC curves of our model (red curve), ISAN model (orange curve), and ICH-A model (green curve) and ICH-B models (blue curve) of SAP in the validation cohort ROC: receiver operating characteristic, AUC: area under the curve; **B**, Decision curves of our model (red curve), ISAN model (orange curve), and ICH-A model (green curve) and ICH-B models (blue curve) in the validation cohort DCA: decision curve analysis

To evaluate the discrimination of the prediction model, we validated the AUROC of our model in the validation cohort and compared it to the ISAN model [7], ICH-APS-A [11] and ICH-APS-B models [11] (Fig. 4A). The AUROC of the validation group (0.8116, 95% CI 0.7499–0.8733) was significantly higher than that of the ISAN and ICH-APS-A/B models (ISAN model: 0.693, 95% CI 0.62–0.766; ICH-A model: 0.7167, 95% CI 0.6448–0.7886; ICH-B model: 0.7228, 95% CI 0.6514–0.7941). Delong’s test revealed a significant difference in AUROC between our model and any of the three previous models (P < 0.05) (Table 3), indicating that our model performs significantly better than these previous models in distinguishing between patients who will develop SAP or not Moreover, our model showed improved performance of reclassification than the ISAN, ICHA and ICHB models, with an NRI of 0.4384 (P < 0.01), 0.5236 (P < 0.01) and 0.4784 (P < 0.01), and an IDI of 0.1279 (P < 0.01), 0.2098 (P < 0.01) and 0.1841 (P < 0.01), respectively (Table 3). Both NRI and IDI quantifies the improvement in a model’s ability to discriminate between two groups or classes (e.g., diseased vs. non-diseased) compared to a baseline or reference model. The NRI calculates the proportion of individuals whose risk classification is improved minus the proportion whose classification is worsened by the new model, compared to the baseline previous models; while the IDI calculates the difference in the average predicted probabilities of the positive and negative classes between the two models. Therefore, these results indicated that our model had a substantially improvement in predictive accuracy over previous models.

**Table 3:** Performance metrics

	ISAN	p-value	ICH-A	p-value	ICH-B	p-value	WMU
AUROC	0.6930 (0.62–0.766)	< 0.001#	0.7167 (0.6448–0.7886)	0.0014#	0.7228 (0.6514–0.7941)	0.0026#	0.8116 (0.7499–0.8733)
NRI	0.4384 (0.2877–0.589)	< 0.001	0.5236 (0.4069–0.6402)	< 0.001	0.4784 (0.346–0.6107)	< 0.001	Reference
IDI	0.1279 (0.0834–0.1724)	< 0.001	0.2098 (0.1519–0.2678)	< 0.001	0.1841 (0.1279–0.2402)	< 0.001	Reference

AUROC: area under the receiver operating characteristic curve; DCA: decision curve analysis; NRI: net reclassification index; IDI: integrated discrimination index;

#: results of Delong’ test compared to our model

In DCA (Fig. 4B), the curve for our model showed a positive net benefit for the threshold probabilities between 10% and 73% compared to the strategies of assuming that all or none of the patients had SAP (i.e., treat-all or treat-none strategies). Moreover, the net benefit of our model is significantly better than any of the three previous models within the threshold probabilities of 10 − 73%.

## Discussion

In this study, we developed a simple, valid, and clinically useful model to predict the probability of developing SAP for individual patients with ICH. This nomogram model incorporated simple risk factors, including male gender, older age, symptoms of dysphagia, disturbance of consciousness, weaker muscle strength, ICH with multilobar involvement and extension into ventricles. All the variables are easy to collect immediately after diagnosis of ICH. With this model, clinicians can quickly calculate the risk of individual patients developing SAP, which may help with effective management decisions. Thus, those with a high risk of developing SAP may benefit from more intensive care, preventative interventions, and earlier treatment.

Several risk factors for developing SAP have been identified in previous studies. Among them, older age is a well-recognized risk factor for pneumonia [24] and has been consistently found in most of the studies for SAP, either in ischemic or hemorrhagic stroke [10, 11]. This could be attributed to the gradual decline in people’s immune function with increasing age, making older individuals more susceptible to infections, including SAP [25]. Male sex is included in our and the ISAN model but not the ICH-APS model. This is probably because current smoking and excess alcohol consumption, which were more frequent in males, are already incorporated as risk factors in the ICH-APS model [7, 11]. Pre-stroke dependence is consistently included in the ISAN and ICH-APS model models, although they were defined as ≥ 2 in the ISAN model [7] while as ≥ 3 in the ICH-APS model [11]. In this study, although more patients with SAP had pre-stroke dependence (mRS ≥ 2), it was not included in the final model after regression analysis. A possible explanation might be that we directly included ‘total muscle strength of the worse side’ as a variable, which reflects not only the affected limbs of the index stroke but also the pre-stroke disability.

Regarding variables associated with stroke symptoms, compared to the ISAN and ICH-APS model, which included an overall NHISS score, our model independently included muscle strength (represented as total muscle strength of the worse side), dysphagia and disturbance of consciousness. We chose the three variables because they were theoretically critical neurological signs associated with SAP as both decreased limb muscle strength and disturbed consciousness can impair the patient’s ability to change positions, leading to pulmonary fluid retention and accumulation of secretions and so increasing the risk of pneumonia, while dysphagia may result in aspiration and increase the risk of SAP. They were consistently recognized as risk factors in previous studies for SAP as well [11, 26, 27]. In addition, they are simpler to evaluate and have a lower collinear relationship to the other variables compared to the NHISS score.

As an ICH-specific characteristic, ‘extension into ventricles’ is a significant and independent factor contributing to both morbidity and mortality [28] and its inclusion as a risk factor in both ICH-APS model and ours is not surprising. As for other hemorrhage-specific characteristics, the hematoma volume is included in the ICH-APS-B model, while multilober involvement is in our model. To some extent, these two variables are similar, as a hemorrhage involving multiple lobes usually have a larger volume than those within a lobe. As for ‘multilobe involvement’, a hemorrhage involving multiple lobes usually have a larger volume than those within a lobe. Regarding the mechanism, this was probably associated with decreased degree of systemic immune regulation due to a large hematoma [29]. However, compared to calculating the volume of ICH, multilobe involvement is easier to determine.

Except for convenience, the more important aim of our study is to improve the model performance for the prediction of SAP compared to existing SAP prediction models developed for ICH. As for the calibration, our model and ISAN model obtained good agreement between observed outcomes and predictions, while the ICH-APS-A and ICH-APS-B models were not well calibrated with significant underestimation. Regarding discrimination, our nomogram model performed significantly better than the ISAN and ICH-APS-A/B models, with an AUROC of 0.8116 versus 0.6930 and 0.7167/0.7228 in our validation sample. As AUROC may not be the optimal measure for evaluating models aimed at predicting future risk or stratifying individuals into risk categories [30] and not sensitive in comparing models with a well-performed baseline model [31], we used NRI and IDI as complement measures to test the discrimination ability [32, 33]. The results further supported that our model offered significant improvement over previous models. Although a model with better discrimination and calibration has theoretically been a better guide to clinical management [34], they are not enough to evaluate whether the prediction model improves clinical decision-making. Therefore, we conducted DCA to assess the clinical decision-making ability of the models [35]. As a result, our model demonstrated a positive net benefit for predicted probability thresholds between 10% and 73%. All these analyses further demonstrated that our model was a more accurate prediction model to identify patients with ICH at higher risk of developing SAP. This allows healthcare providers to tailor the allocation of medical resources to take more intensive care or interventions for these high-risk patients to prevent the progression of SAP or reduce its severity, thus contributing to an overall improved prognosis of the population with ICH.

Our study had several limitations. First, our study included only patients admitted into the neurology department, and those who died in the emergency department or were admitted to the intensive care unit were not included. This will inevitably result in selection bias and the underestimation of outcome events (SAP). Therefore, our model is more applicable to ICH patients with mild to moderate stroke severity compared to those with severe conditions. However, this problem also existed in the previous studies, as the median of NIHSS score of the study subjects was 9 for the ICH-APS-A/B models (IQR 3–16) and 4 for the ISAN model (IQR 2–9). Although the severity of our study population is comparable to these previous studies, caution should be exercised when interpreting the results due to the common selection bias in the patient populations of all three studies. Further studies should be conducted to validate the predictive performance of the models for those with severe stroke. Second, as a retrospective study, 12 patients were excluded from the study due to without an initial CT performed within 72 h post-ICH. This might also lead to the objective existence of selection bias. Third, other clinical data not considered in the existing model may have a confounding effect and impact the pneumonia risk. For example, the patient’s medication history, such as use of glucocorticoids or immunosuppressive drugs, may result in an immunosuppressive state and influence the risk of developing SAP. Lastly, this is a single-center study with internal verification conducted on patients in a tertiary hospital. Therefore, it is not clear whether the model can be applied to external patients, especially those from secondary and primary healthcare institutions. To address these limitations, in future investigations, we should carry out a large-sized prospective study, including not only patients admitted to the neurology department but also those who experienced mortality in the emergency department or were admitted to the intensive care unit, and conduct external validation at both secondary and primary healthcare institutions.

## Conclusions

In summary, male patients with older age, multilobar involvement, the extension of ICH into ventricles, dysphagia, disturbance of consciousness, and worse muscle strength were at higher risk of developing SAP following ICH. The nomogram model obtained in this study is convenient and shows better predictive performance than previous models. Therefore, it might be a promising tool to assess the individual risk of developing SAP for patients with ICH and thus facilitate preventive measures. Nevertheless, a further improved prediction model can be achieved through a larger-sized well-designed prospective research with external validation in the future.

### Electronic supplementary material

Below is the link to the electronic supplementary material.

Supplementary Material 1

## Data Availability

The datasets used and/or analysed during the current study are available from the corresponding author on reasonable request.
