# Supplementary material for: Development and validation of a nomogram model for prediction of stroke-associated pneumonia associated with intracerebral hemorrhage
Source: BMC Geriatr. 2023 Oct 7;23:633. doi: 10.1186/s12877-023-04310-5 (PMC10559607; doi:10.1186/s12877-023-04310-5)
Supplement: Supplementary file 1 — Supplementary Material 1 [file 12877_2023_4310_MOESM1_ESM.docx]

**Supplementary Table 1. Collinearity detection of candidate variables.**

| **Variables** | **Variables** | **Variance inflation factor (VIF)** |
| --- | --- | --- |
| Age (years), median (IQR) | 0.827 | 1.209 |
| Sex (male), No./total No. (%) | 0.579 | 1.728 |
| Smoking, No./total No. (%) | 0.637 | 1.569 |
| Alcohol drinking, No./total No. (%) | 0.671 | 1.491 |
| Hypertension, No./total No. (%) | 0.957 | 1.044 |
| Diabetes, No./total No. (%) | 0.731 | 1.367 |
| Hyperlipidemia, No./total No. (%) | 0.958 | 1.044 |
| Ischemic heart disease, No./total No. (%) | 0.969 | 1.032 |
| Hyperuricemia, No./total No. (%) | 0.918 | 1.089 |
| COPD, No./total No. (%) | 0.955 | 1.047 |
| Prestroke dependence (mRS≥2), No./total No. (%) | 0.964 | 1.038 |
| Dysphagia, No./total No. (%) | 0.588 | 1.701 |
| Disturbance of consciousness, No./total No. (%) | 0.439 | 2.279 |
| Total muscle strength of worse side, median (IQR) | 0.274 | 3.651 |
| Vomiting after ICH, No./total No. (%) | 0.879 | 1.138 |
| NIHSS | 0.145 | 6.899# |
| GCS | 0.284 | 3.522 |
| Multilobar involvement, No./total No. (%) | 0.837 | 1.195 |
| Deep region involvement, No./total No. (%) | 0.792 | 1.262 |
| Extension into ventricles, No./total No. (%) | 0.934 | 1.071 |
| Lesion volume (ml), median (IQR) | 0.815 | 1.226 |
| Red blood cell (10^12^/L), median (IQR) | 0.79 | 1.266 |
| Platelet (10^9^/L), median (IQR) | 0.925 | 1.081 |
| Albumin, median (g/L) (IQR) | 0.973 | 1.028 |
| Blood glucose (mmol/L), median (IQR) | 0.693 | 1.443 |
| Creatinine (μmol/L), median (IQR) | 0.916 | 1.092 |

#VIF > 5 was considered the existence of collinearity

**Supplementary Table 2 ISAN model[1]**

| Item | Score |
| --- | --- |
| Age, y |  |
| <60 | 0 |
| 60 to 69 | 3 |
| 70 to 79 | 4 |
| 80 to 89 | 6 |
| 90+ | 8 |
| Sex |  |
| Female | 0 |
| Male | 1 |
| NIHSS on admission |  |
| 0 to 4 | 0 |
| 5 to 15 | 4 |
| 16 to 20 | 8 |
| 21+ | 10 |
| mRS prestroke |  |
| Independent | 0 |
| Not independent | 2 |

**Supplementary Table 3 ICH-APS-A/B models[2]**

| Items | Score | |
| --- | --- | --- |
|  | ICH-APS-A | ICH-APS-B |
| Age group |  |  |
| ≤59 | 0 | 0 |
| 60-69 | 2 | 2 |
| 70-79 | 3 | 3 |
| ≥80 | 5 | 5 |
| Current smoking |  |  |
| No | 0 | 0 |
| Yes | 1 | 1 |
| Excess alcohol consumption |  |  |
| No | 0 | 0 |
| Yes | 1 | 1 |
| COPD |  |  |
| No | 0 | 0 |
| Yes | 5 | 6 |
| Pre-stroke dependence (mRS≥3) |  |  |
| No | 0 | 0 |
| Yes | 2 | 2 |
| Admission GCS score |  |  |
| 15 | 0 | --- |
| 13-14 | 2 | --- |
| 9-12 | 2 | --- |
| 3-8 | 2 | --- |
| Admission NIHSS score |  |  |
| 0-5 | 0 | 0 |
| 6-10 | 1 | 2 |
| 11-15 | 2 | 3 |
| ≥16 | 3 | 5 |
| Dysphagia |  |  |
| No | 0 | 0 |
| Yes | 2 | 3 |
| Infratentorial location |  |  |
| No | 0 | 0 |
| Yes | 1 | 1 |
| Extension into ventricles |  |  |
| No | 0 | --- |
| Yes | 1 | --- |
| Hematoma volume (ml) |  |  |
| infratentorial ICH <10 or supratentorial ICH <40 | --- | 0 |
| infratentorial ICH 10-20 or supratentorial ICH 40-70 | --- | 1 |
| infratentorial ICH >20 or supratentorial ICH>70 | --- | 2 |

**References**

1. Smith CJ, Bray BD, Hoffman A, Meisel A, Heuschmann PU, Wolfe CD, Tyrrell PJ, Rudd AG, Intercollegiate Stroke Working Party G: **Can a novel clinical risk score improve pneumonia prediction in acute stroke care? A UK multicenter cohort study**. *J Am Heart Assoc* 2015, **4**(1):e001307.

2. Ji R, Shen H, Pan Y, Du W, Wang P, Liu G, Wang Y, Li H, Zhao X, Wang Y *et al*: **Risk score to predict hospital-acquired pneumonia after spontaneous intracerebral hemorrhage**. *Stroke* 2014, **45**(9):2620-2628.
